# Variants in Nebulin (*NEB*) Are Linked to the Development of Familial Primary Angle Closure Glaucoma in Basset Hounds

**DOI:** 10.1371/journal.pone.0126660

**Published:** 2015-05-04

**Authors:** Dina F. Ahram, Sinisa D. Grozdanic, Helga Kecova, Arjen Henkes, Rob W. J. Collin, Markus H. Kuehn

**Affiliations:** 1 Department of Ophthalmology and Visual Sciences, The University of Iowa, Iowa City, IA, United States of America; 2 Animal Eye Consultants of Iowa, North Liberty, IA, United States of America; 3 Department of Human Genetics, Radboud University Medical Center, Nijmegen, The Netherlands; 4 Radboud Institute for Molecular Life Sciences, Radboud University Medical Center, Nijmegen, The Netherlands; Casey Eye Institute, UNITED STATES

## Abstract

Several dog breeds are susceptible to developing primary angle closure glaucoma (PACG), which suggests a genetic basis for the disease. We have identified a four-generation Basset Hound pedigree with characteristic autosomal recessive PACG that closely recapitulates PACG in humans. Our aim is to utilize gene mapping and whole exome sequencing approaches to identify PACG-causing sequence variants in the Basset. Extensive clinical phenotyping of all pedigree members was conducted. SNP-chip genotyping was carried out in 9 affected and 15 unaffected pedigree members. Two-point and multipoint linkage analyses of genome-wide SNP data were performed using Superlink-Online SNP-1.1 and a locus was mapped to chromosome 19q with a maximum LOD score of 3.24. The locus contains 12 Ensemble predicted canine genes and is syntenic to a region on chromosome 2 in the human genome. Using exome-sequencing analysis, a possibly damaging, non-synonymous variant in the gene Nebulin (*NEB*) was found to segregate with PACG which alters a phylogenetically conserved Lysine residue. The association of this variants with PACG was confirmed in a secondary cohort of unrelated Basset Hounds (p = 3.4 × 10^-4^, OR = 15.3 for homozygosity). Nebulin, a protein that promotes the contractile function of sarcomeres, was found to be prominently expressed in the ciliary muscles of the anterior segment. Our findings may provide insight into the molecular mechanisms that underlie PACG. The phenotypic similarities of disease presentation in dogs and humans may enable the translation of findings made in this study to patients with PACG.

## Introduction

Glaucoma is an optic neuropathy and the leading cause of blindness worldwide [1]. Primary angle-closure glaucoma (PACG) is the second most common form of glaucoma after primary open-angle (POAG) and is three times more prevalent than POAG in Chinese, Asian Indian and Eskimo populations [2,3]. The condition affects 9.4 million people in China alone where it is estimated to be responsible for the vast majority of bilateral glaucoma blindness cases [4].

All forms of glaucoma are characterized by progressive and irreversible damage of the optic nerve (ON) and degeneration of retinal ganglion cells (RGCs), which result in optic disc cupping and visual field loss [5–7]. Elevated intraocular pressure (IOP) is a primary risk factor for the initiation and progression of both forms of glaucoma [8]. In many cases however, visual field loss is still noted despite adequate control of elevated IOP, which suggests the action of additional molecular mechanisms in the pathogenesis of glaucoma, which are yet to be identified [9].

The hallmark of PACG is the collapse of the iridocorneal angle due to the anterior movement of the iris, which results in the obstruction of aqueous humor drainage at the trabecular meshwork [10]. In addition to increased IOP, anatomical risk factors for PACG include, narrowing of the iridocorneal angle, shallowness of the central and/or peripheral anterior chamber as well as reduced axial length of the globe [11,12]. Additionally, older age and female gender have been identified as disease risk factors [13–15]. Despite recognition of disease risk factors, the underlying genetic and environmental contributors to the development and progression of PACG have not been fully defined.

In addition to humans, glaucoma has also been reported in the dog (Gelatt and MacKay, 2004, Grozdanic et al., 2010). Examination of animals in teaching hospitals revealed the highest prevalence of glaucomas in the American Cocker Spaniel (5.52%) and Basset Hound (BH) (5.44%) among several other dog breeds. An overall predominance of disease presentation is also noted in females versus males [16,17]. A total of 5.44% of all Bassets treated in the clinic presented with glaucoma, representing a significantly higher fraction than most breeds [17]. In BH, the most common form of angle occlusion occurs as a result of forward movement of the iris towards the cornea when significant posterior-to-anterior pressure difference develops, thus, collapsing the ciliary cleft and compressing the TM [18]. Unfortunately, treatment options are limited and affected animals often develop bilateral blindness. Due to its impact on multiple breeds presenting with comparable phenotypes, the identification of a PACG causing gene in the BH may serve to determine the risk of disease in other breeds possibly sharing the same mutation.

The role of genetics has also been demonstrated in dog glaucoma. In a recent GWA study of PACG that we conducted in a Basset Hound cohort, we identified two susceptibility loci on chromosome 14 (*COL1A2*) and chromosome 24 (*RAB22A*) [19]. Furthermore, using a family-based mapping approach, a non-synonymous substitution in the metalloproteinase gene (*ADAMTS10*) was identified in a Beagle pedigree affected with POAG [20].

Here, we have utilized both linkage analysis as well as homozygosity mapping to investigate and identify the genetic mechanisms underlying PACG in a pedigree of Basset Hounds identified with segregating PACG. The large number of genetically informative affected animals in this pedigree provides sufficient statistical power for the mapping of a genetic locus that segregates with the disease phenotype in the affected animals.

## Materials and Methods

### Animals and Clinical Presentation

**Fig 1:**
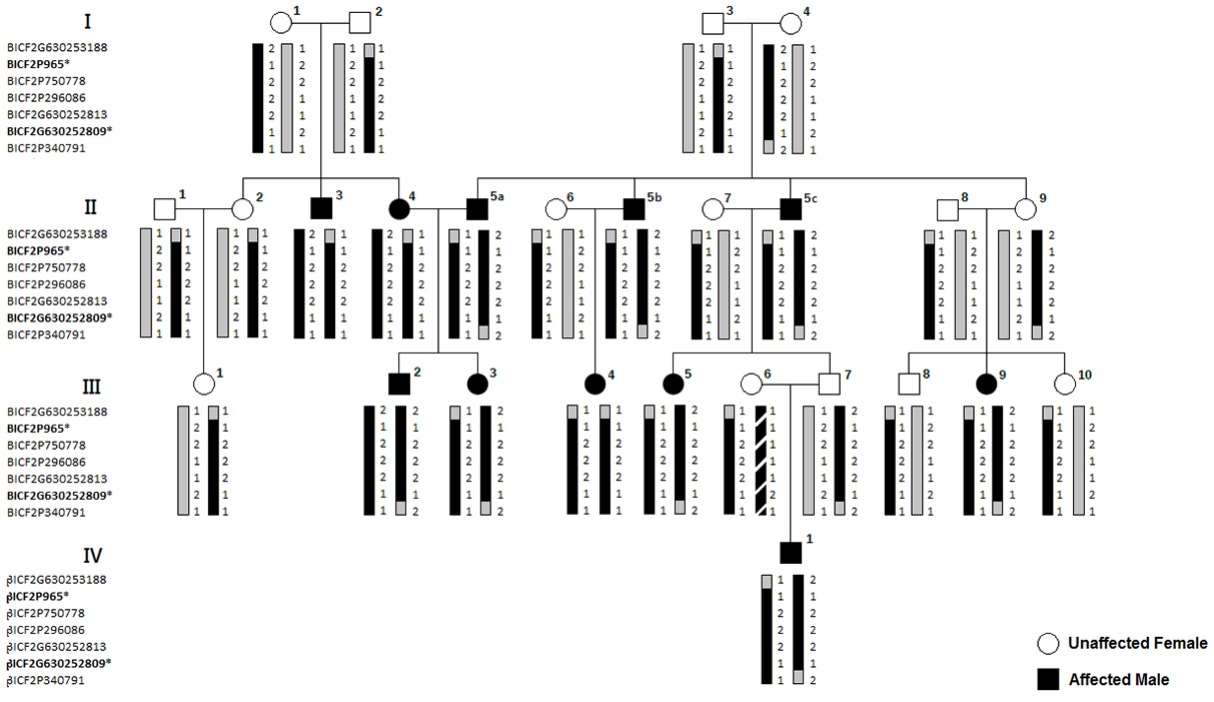
Basset Hound Pedigree used in this study. The affected Basset 5a in the second generation was duplicated twice (5b and 5c) in order to break two otherwise computationally confounding breeding loops. Genotypes of typed markers within region uncovered following two-point linkage analysis are shown. Shading indicates the transmission pattern of heterozygous parental haplotypes to affected, homozygous offspring. Additional patterns of shading including diagonal lines indicate variation to the same haplotype identified in the other pedigree members. Complete concordance of homozygous haplotype inheritance with the disease phenotype is observed in all affected animals.

Genome wide linkage analysis and homozygosity mapping studies were conducted using affected and unaffected Basset Hounds derived from the pedigree shown in Fig 1. With the owners’ consent whole blood was collected from 24 Basset Hound pedigree members (9 affected, 15 unaffected) as well as 44 additional animals which were unrelated to the primary pedigree. The animals from the second cohort were derived from several pedigrees including: pedigree 2 (5 affected, 6 unaffected), pedigree 3 (3 affected, 2 unaffected), pedigree 4 (2 affected, 2 unaffected) and unrelated animals (16 affected, 8 unaffected). All animals utilized in this study were derived from Basset Hound breeders residing in Northern America.

All animal studies were conducted in accordance with the ARVO statement for use of animals in ophthalmic and vision research. Procedures conducted were approved by the respective University of Iowa and Iowa State University Committees on Animal Care and Use. All animals included were examined by a veterinary ophthalmologist as previously described [18]. Affected animals were diagnosed with PACG following a thorough clinical evaluation. Examination of the anterior segment of all dogs used in this study included gonioscopy, slit lamp microscopy (Kowa SL-15, Optimed Inc., Torrance, CA, USA), and high-frequency ultrasonography (HRUS) with a 35-MHz probe (E-Technologies, Bettendorf, IA).

Animals with possible secondary glaucoma or other confounding ocular conditions were excluded from the study. To ensure accurate diagnosis of their status, unaffected dogs were at least four years of age, and had normal iridocorneal angle and cleft conformation observed by gonioscopy and high-resolution ultrasound.

Human eyes (N = 2) from unaffected human donors were obtained with the consent of the next of kin for immunofluorescence investigation within 6 hours postmortem. Cadavers are not human subjects and therefore institutional review board approval was not required. Additionally, (N = 2) eyes were collected from unaffected Basset Hounds as well as (N = 2) eyes from Basset Hounds that underwent enucleation due to clinical glaucoma refractive to medical and/or surgical treatment were submitted for histopathology and histochemical investigation.

### SNP Genotyping, Linkage Analysis and Homozygosity Mapping

Genomic DNA was extracted from Ethylenediaminetetraacetic acid (EDTA)-stabilized whole blood samples using the Qiagen DNeasy blood and tissue kit (Qiagen, Hilden, Germany). DNA samples were eluted with de-ionized water and stored at -20°C. Samples were genotyped using the Illumina CanineHD BeadChip (Illumina, San Diego, CA), which contains 172, 000 markers placed on a CanFam2.0 reference sequence. SNP genotypes and Mendelian errors were removed from all genotyped samples using a missing genotype rate of < 5% and a SNP call rate > 95% threshold. Two-point and multipoint linkage analyses of genome wide SNP data were performed using Superlink-Online SNP-1.1 [21]. A logarithm of odds (LOD) score of 3 or higher was used to establish statistically significant linkage. A recessive model was specified with a susceptibility allele frequency of 0.01, a penetrance value of 0.99 and an assumption of no phenocopies. A maximum-likelihood haplotype configuration that takes into account intermarker recombination fractions was estimated for markers within the region of linkage using the software Superlink-Online SNP-1.1 [21]. Homozygosity mapping was performed using the online tool Homozygosity Mapper-Dog [22].

### Whole Exome Sequencing Analysis

Canine exome sequencing was performed following protocols similar to human exome sequencing [23]. In brief, enrichment of exonic sequences was achieved by using the SureSelect^XT^ Canine All Exon Kit (Agilent, Santa Clara, CA, USA). Massive parallel sequencing of genomic DNA from each of the ten canine exome libraries was performed using the SOLiD 5500XL platform (Life Technologies, Foster City. CA, USA). On average, we obtained more than 113 million mappable sequencing reads (50bp single-end) and ~5.5 Gb of mappable sequence data per individual dog sample after multiplex sequencing. Color space reads were mapped to the CanFam2.0 canine reference genome with SOLiD LifeScope software version 2.1, which uses an iterative mapping approach. Single-nucleotide variants were subsequently called by the DiBayes algorithm using high-stringency calling settings.

### Generation of Annotation Pipeline

To annotate all sequence variants and predict their effect at the DNA, RNA and protein level, an automated pipeline was build. Due to the poor annotation of the Canfam2.0 canine reference genome, four different gene prediction programs (Gnomon, N-scan, Ensembl and human genes mapped to the canine genome) were used to annotate canine genes. Following sequence reads mapping, an automated pipeline was used to identify the nature of all variants, including the predicted effect on the DNA, RNA and protein level, a bio-informatic prediction of pathogenicity based on SIFT [24] and PolyPhen [25], information on the human reference amino acid at the orthologous position as well as OMIM and gene ontology information.

### Sequencing of Candidate Genes and Mutation Screening

Sanger sequencing validation of variants identified within the two candidate genes was performed with primer sets designed using Primer 3 program (http://bioinfo.ut.ee/primer3-0.4.0/) (Table 1). Primers were designed to bracket variants identified within coding regions and were validated using NCBI Primer-BLAST (http://www.ncbi.nlm.nih.gov/tools/primer-blast/) to ensure their specificity. All primers were designed using the Canine genomic reference sequence (CanFam2.0) (http://www.broadinstitute.org/mammals/dog). Sequencing of PCR products was conducted with BigDye Terminator using the ABI 3100 sequencing platform (Applied Biosystems, Foster City, USA). Sequencing reaction chromatograms were analyzed using DNA Baser sequence assembly software (Pitesti, Romania).

**Table 1:** PCR primer sequences and reaction conditions.

Gene	Variant Details	Primer Sequences	Fragment Size (bp)	Annealing Temp (°C)
*RIF1*	g.55723957 C->T	F'-GAATCTGAAGCGGAGACAGC	285	55
		R'-TTCACTGCTCACCTCACCAA		
*NEB*-1	g.55856370 C->A	F'-TCCATGCATGTGGCCAAG	340	57
		R'-ATGTTATTAATTCTGAGGGCTCCA		
*NEB*-2	g.55856628 G->C	F'-GAGATAAGTTGATATTTGGATGATTCC	348	57
		R'-CTAAGGTCTGGCACTTCTTGG		
*NEB*-3	g.55885214 A->G	F'-TGGAAATGATGTCACAGTGCT	285	58
		R'-TGTTTGCAAAATTCATCCCTTA		

Targeted sequencing of the genomic variant g.55723957 C->T in the gene *RIF1* and g.55885214 A->G in the gene *NEB* was performed in a confirmation cohort comprised of 44 unrelated and related animals detailed in the animals and clinical presentation section.

### Bioinformatic and *In-silico* Analyses

All missense mutations identified were analyzed using the *in silico* pathogenicity prediction tools SIFT (Sorting Intolerant From Tolerant) (http://sift.bii.a-star.edu.sg) and PolyPhen-2 version 2.2.2, (http://genetics.bwh.harvard.edu/pph/) [24,26,27]. Polyphen-2 was additionally used to establish the conservation profile of the nebulin protein sequence among various species by integrating the UCSC Genome Browser’s human genome annotations and MultiZ multiple alignments of vertebrate genomes. Protein domain identification in Nebulin was performed using the tool SMART (a Simple Modular Architecture Research Tool) (http://smart.embl-heidelberg.de) [28]. Investigation of the location of Nebulin amino acid variants within protein structural domains was conducted following the identification of the Nebulin secondary structure.

### Immunohistochemical Analysis of Dog Eyes

Eyes derived from two affected and two unaffected animal were fixed in 4% paraformaldehyde immediately following enucleation. Tissues were dehydrated and paraffin embedded using standard procedures. 7μm sagittal sections were made using a rotary microtome. Tissue sections were deparaffinized in xylene for 20 minutes, then hydrated in decreasing ethanol concentrations for 5 minutes at each concentration. Heat-assisted antigen retrieval was performed using 10mM citrate buffer (pH 6.0) for 10 minutes at 95–100°C. Blocking of endogenous peroxidase activity was achieved by treating sections with 2% H_2_O_2_-methanol solution for 20 minutes at room temperature. Sections were blocked in 5% normal goat serum /1% bovine serum albumin solution in 1X TBS for 1 hour at room temperature then incubated overnight at 4°C in a polyclonal primary rabbit anti-Nebulin (H-300) (Santa Cruz Biotechnology, Inc. Dallas, TX) diluted at a concentration of 1:150 in 1X TBS. A negative control was generated by omitting the primary antibody. All sections were treated with biotinylated secondary anti-rabbit IgG antibody (1:100) (Vector, Burlingame, CA). Color development was achieved using an Avidin/Biotinylated—peroxidase Complex VECTASTAIN ABC kit (Vector, Burlingame, CA) by applying directly to tissue sections for 1 minute. Sections were dehydrated, cleared in xylene and mounted using Permount mounting media (Thermo Fisher Scientific Inc, Waltham, MA). Immunohistochemical staining was evaluated using light microscopy (Olympus BH-2, Center Valley, PA).

### Immunofluorescence Analysis of Human Donor Eyes

Donor eyes derived from 2 unaffected individuals were fixed within 6 hours of death in 4% paraformaldehyde for 2 hours at room temperature. Fixed tissues were cryoprotected in 50% sucrose, embedded in OCT and 7μm sections were cut on a cryostat. After washing with phosphate-buffered saline (PBS) solution, sections were incubated in 1X PBS containing 1% bovine serum albumin for 45 minutes at room temperature to block background signals. Sections were incubated in with the primary Nebulin Antibody as described above. A control was generated by omitting the primary antibody. After washing, all sections including the negative control were incubated with Alexa Fluor 488 goat secondary anti-rabbit IgG (H+L) (Life Technologies, Grand Island, NY) diluted at 1:200 in a dapi-1X PBS solution (1:25,000), for 1 hour at room temperature. Sections were rinsed and cover-slipped using aqua mount mounting medium (Thermo Scientific, USA) then examined using fluorescence microscopy at 488nm.

## Results

### Animals and Clinical Presentation

Our genetic investigation of familial PACG included 22 Basset Hounds, including 9 affected and 13 unaffected dogs that were derived from a single, four-generation pedigree (Fig 1). As previously shown, the development of elevated IOP and subsequent vision loss is correlated with the gradual narrowing of the iridocorneal angle, resulting in angle closure in all affected animals [18]. In concordance with these findings, all affected pedigree members included in this study displayed completely collapsed iridocorneal angles and ciliary clefts confirmed using extensive HRUS and gonioscopy examination. Angle and cleft collapse were observed in association with notable IOP elevation of at least 25 mmHg in all affected animals. All unaffected pedigree members displayed normal IOPs, and had normal appearing iridocorneal angles by gonioscopy and HRUS.

### SNP Genotyping, Linkage Analysis and Homozygosity Mapping

**Fig 2:**
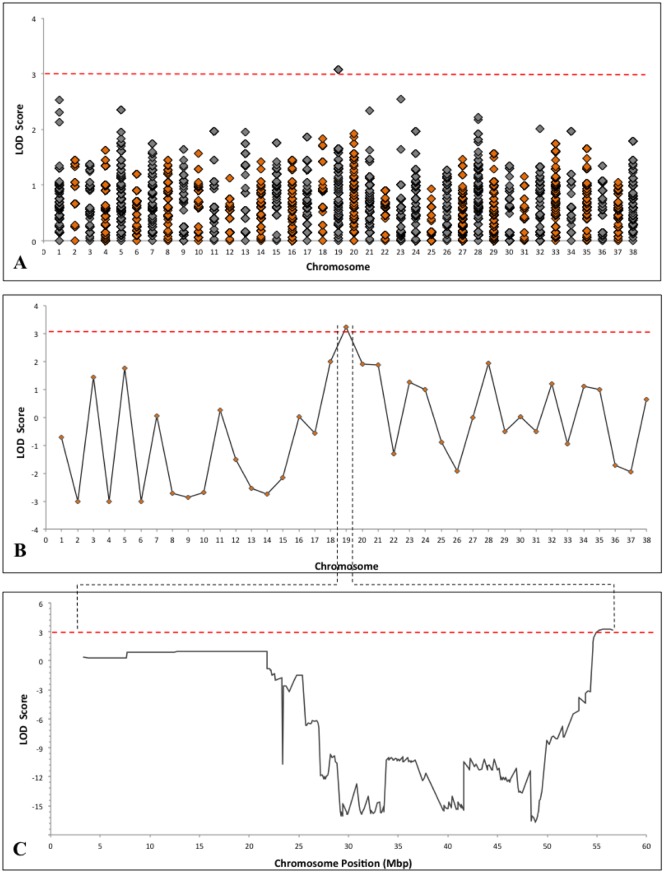
Genome-wide linkage analysis of SNP genotype data. Using two-point linkage analysis, a maximum LOD_two-point_ score of 3.07 was achieved for a 0.49 Mb locus on chromosome 19. The red line of statistical significance indicates LOD scores values > 3 (A). Using multipoint linkage analysis, an increased maximum LOD_multipoint_ score of 3.24 was achieved for a locus mapped to the same location (B). A schematic view of the maximum LOD scores achieved across chromosome 19. The statistically significant locus is located at the distal end of chromosome 19 (Chr19: 54,949,124–56,765,346) and spans 1.82 Mbp (C).

**Fig 3:**
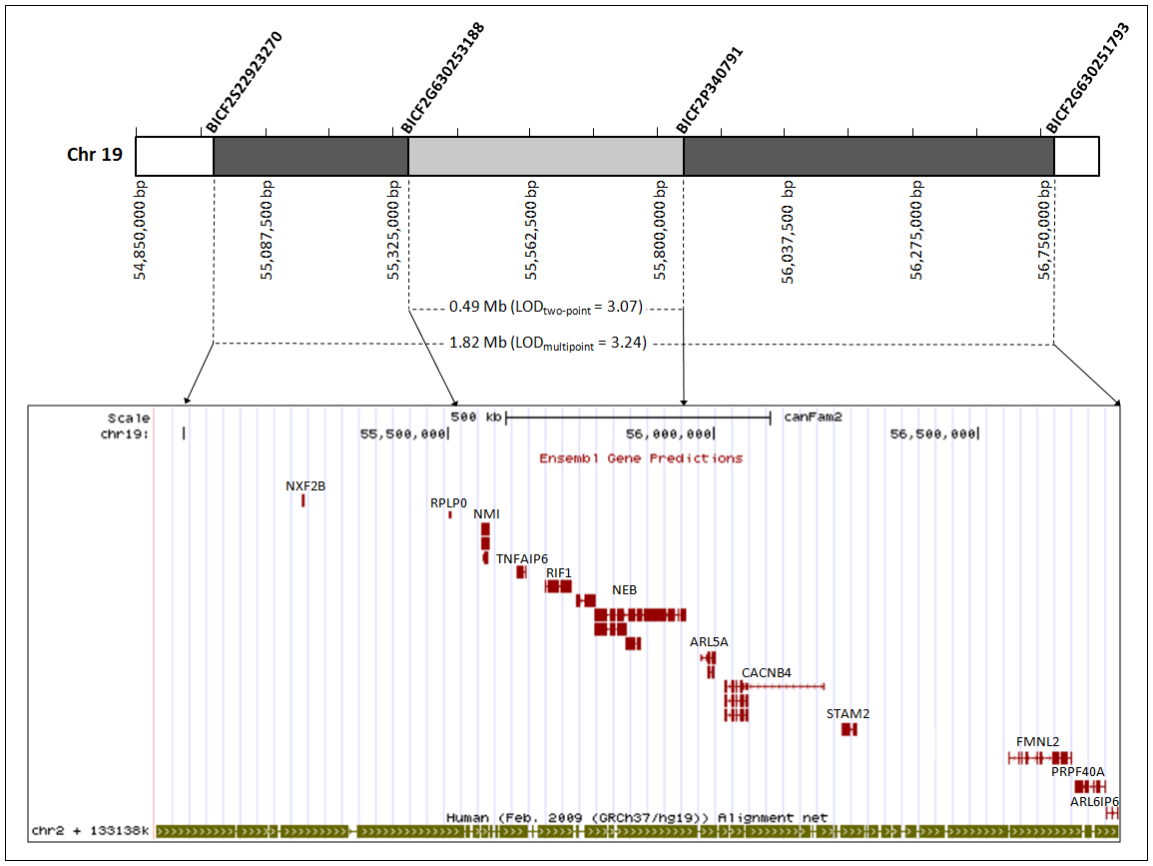
Graphical representation of two-point and multipoint linkage analysis results. The larger locus identified with using multipoint linkage analysis a maximum LOD_multipoint_ score of 3.24 (Chr19: 54,949,124–56,765,346) brackets the region identified using two-point linkage analysis (Chr19: 55,358,186–55,848,473) and contains a total of 12 Ensemble-predicted canine genes. Comparison of the linked locus to the human genome reveals shared synteny with a region on chromosome 2. The number and order of genes identified within the canine locus are completely conserved in the syntenic human region.

Using genome-wide, two-point linkage analysis, a 0.49 Mb region was mapped to the distal portion of chromosome 19 (Chr19: 55,358,186–55,848,473) with a maximum LOD score of 3.07 (Fig 2A). The identified locus is represented by 41 probe sets. Investigation of the phase and inheritance pattern of the identified haploblock revealed complete concordance of the locus inheritance with the disease phenotype in all affected versus unaffected carriers. Multipoint linkage analysis extended the locus to 1.82 Mbp (Chr19: 54,949,124–56,765,346) and achieved a LOD score of 3.24 (Fig 2B and 2C). This extended locus is represented by 151 probe sets and contains a total of 12 Ensemble-predicted canine genes (Fig 3). A theoretical maximum LOD score was manually calculated for this pedigree and was estimated to be 5.4.

**Fig 4:**
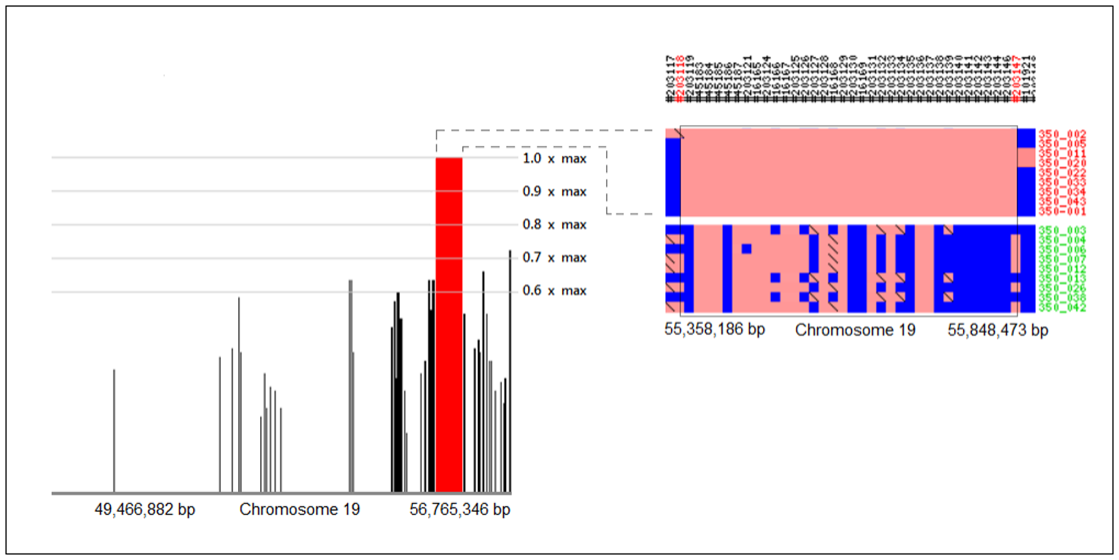
Whole genome homozygosity mapping results. A red peak reaching a 1.0 max score of statistical significance depicts a homozygous region on chromosome 19. The region coincides with the 0.49 Mbp locus identified using two-point linkage analysis and is bracketed by recombination spots (indicated in red). The identified haploblock fulfills the zygosity criterion by displaying homozygosity in affected animals (indicated in red) and heterozygosity in unaffected carriers (indicated in green).

Analysis of SNP genotype data using homozygosity mapping revealed sharing of the 0.49 Mbp two-point linkage locus among all affected pedigree members (Fig 4). The locus fulfills the hypothesized zygosity criterion by displaying homozygosity in affected animals and heterozygosity in unaffected carriers. The homozygous locus is flanked by the markers BICF2G630253188 and BICF2P340791 and contains the four Ensemble-predicted canine genes *NMI*, *TNFAIP6*, *RIF1* and *NEB*. The results of homozygosity mapping support the findings of the two-point genetic linkage analysis (Figs 3 and 4).

### Whole Exome Sequencing and Variant Screening

Exome sequencing was carried out an affected dog (Fig 1, number 5) and the two unaffected parents (numbers 3 and 4). We identified the total number of variants for every exome sequenced and determined the fraction of missense variants among all other variants identified, including variants in coding and non-coding sequences (Table 2). 170 substitutions were found to conform to our criteria of zygosity (criteria matched substitutions), i.e. homozygous in the affected animals and heterozygous in the unaffected carriers. We also determined the total number of nonsense mutations in every exome. However, no nonsense mutations adhering to the criteria of zygosity were identified.

**Table 2:** Whole exome-capture sequencing analysis.

	Sample Name	Affection Status	Average Exome Coverage	Total Bases	Mapped Reads	Total Variants	Missense Variants	Criteria Matched Substitutions	Nonsense Mutations	Criteria Matched Nonsense Mutations
**Pedigree 1**	1	Unaffected	42	3,844,586,200	36,793,718	111,623	5735	170	9	0
	2	Unaffected	34	3,014,346,400	28,908,927	104,403	6009		13	
	3	Affected	37	3,375,846,000	32,324,874	104,350	5758		13	

Exome sequencing analysis was conducted in a trio derived from the PACG pedigree investigated using linkage analysis. The total number of reads, mapped reads, and the mean coverage for every exome sequenced is shown. In addition, the total number of variants and fraction of missense variants including substitutions found to conform to our criteria of zygosity are displayed.

24 of the criteria matched substitutions were located in exons captured within the 0.49Mb linked region, and four of which were found to result in amino acid substitutions (Table 3). These included a missense variation in the Telomere-Associated Protein RIF1 encoding gene *RIF1* g.55723957 C->T in exon 30 that resulted in an amino acid change at position p.1992 Ala->Val (Transcript ID: ENSCAFT00000009138; Protein ID: ENSCAFP00000008476). This variant is predicted to be benign using functional prediction tools. The three remaining missense variants were identified in the Nebulin protein-coding gene *NEB*. Both g.55856370 C->A in exon 62 (p.2891 Gln->Lys) and g.55856628 G->C in exon 62 (p.2805 Val->Leu) (Transcript ID: ENSCAFT00000009249; Protein ID: ENSCAFP00000008582) are likely benign variants. However, the third *NEB*-based variant g.5588214 A->G in exon 48 (p.2051 Lys->Arg) was predicted to affect protein function and was the focus of further investigation.

**Table 3:** Criteria-matched candidate variants identified in the region of linkage.

Gene	Genomic Change	Amino Acid Change	Function	Functional Prediction	
				PolyPhen-2	SIFT
***RIF1*** (Telomere-Associated Protein RIF1)	g.55723957 C->T	(ENSCAFT00000009138) p.1992 A->V	NonSyn SNV	Benign	Tolerated
***NEB* (**Nebulin)	g.55856370 C->A	(ENSCAFT00000009249) p.2891 Q->K	NonSyn SNV	Benign	Tolerated
	g.55856628 G->C	(ENSCAFT00000009249) p.2805 V->L	NonSyn SNV	Benign	Tolerated
	g.55885214 A->G	(ENSCAFT00000009249) p.2051 K->R	NonSyn SNV	Possibly damaging	Predicted to affect protein function

Criteria of zygosity, genomic position and functional prediction were applied to identify five nonsynonymous variants (NonSyn SNV). A single missense variants was identified in *RIF1*. Three missense vriantss were identified in *NEB*, one of which was predicted to be possibly damaging.

### Sequencing of Candidate Genes and Variant Screening

**Fig 5:**
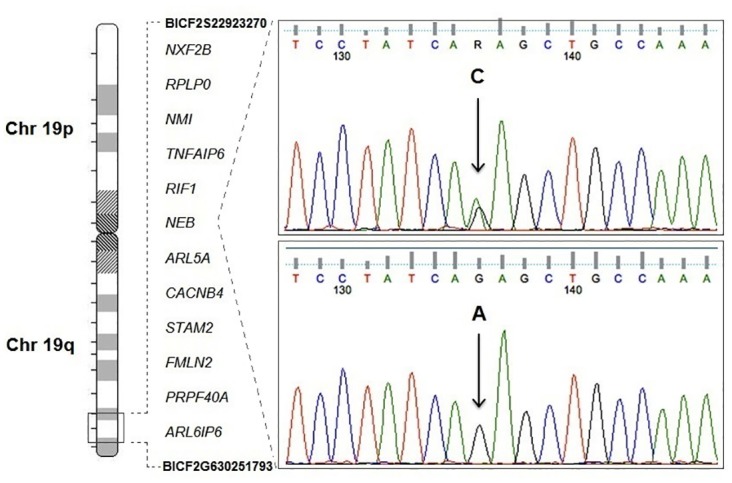
Sanger sequencing confirmation of the disease segregating variant identified in the linked region. Candidate region identified using linkage analysis in PACG pedigree animals (left). Sequence chromatograms from a carrier (C), confirms heterozygous genotype whereas affected animals (A) display a homozygous state of variant identified in *NEB* (g.5588214 A->G) (Right).

Using targeted Sanger sequencing, the segregation of the four non-synonymous variants identified within the region of linkage in the affected versus unaffected pedigree animals was confirmed. Targeted sequencing of (g.55723957 C->T) in *RIF1* and (g.55856370 C->A, g.55856628 G->C, and g.55885214 A->G) in *NEB* was performed in all members of the pedigree (Fig 1), including additional unaffected animals not used during linkage analysis. All affected animals were confirmed to display homozygosity, whereas unaffected animals displayed heterozygosity for all four variants, consistent with autosomal recessive inheritance of the disease-linked haplotype (Fig 5).

The association of the *NEB* variant (g.55885214 A->G) with PACG was confirmed in a second, independent cohort. Sanger sequencing of the 44 animals from this cohort demonstrated the segregation of homozygous GG in 88% and heterozygous AG in 12% of the affected animals sequenced. In contrast, the segregation of homozygous GG, heterozygous AG and homozygous AA was observed in 33.3% and 44.4% and 22.2% of the unaffected animals sequenced respectively. A Fisher exact test statistic P-value of 0.00034 demonstrates statistical significance of the genotype distribution in this second group of animals (Table 4). Similarly the association of the *RIF1* variant (g.55723957 C->T) with PACG was investigated in the same independent cohort. Sanger sequencing of the 44 cohort animals demonstrated a segregation of (TT (6.9%), CT (48.3%), CC (44.8%)) in the affected animals, while in the unaffected animals, a segregation of (TT (0%), CT (40%), CC (60%)) was observed. A Fisher exact test statistic P-value of 0.57 demonstrates a lack of statistical significance for the genotype distribution in this second group of animals as well as the absence of linkage between this variant and the disease in the general population (Table 5).

**Table 4:** A Fisher exact contingency table of genotypes observed in a confirmatory animal cohort for the *NEB* variant (g.55885214 A->G).

	TT	CT	CC	Marginal Row Totals
**Unaffected**	0 (0%)	6 (40%)	9 (60%)	15
**Affected**	2 (6.9%)	14 (48.3%)	13 (44.8%)	29
**Marginal Column Totals**	2	20	22	44 (Grand Total)

Forty-four additional unaffected and affected Basset Hounds were selected for confirmatory sequencing of the *NEB* variant (g.55885214 A->G). The observed total of individuals and percentage of individuals displaying a specific genotype is shown for each cell respectively.

The two-tailed P value is 0.00034 (Fisher Exact Probability Test)

**Table 5:** A Fisher exact contingency table of genotypes observed in a confirmatory animal cohort for the *RIF1* variant (g.55723957 C->T).

	GG	AG	AA	Marginal Row Totals
**Unaffected**	6 (33.3%)	8 (44.4%)	4 (22.2%)	18
**Affected**	23 (88%)	3 (12%)	0 (0%)	26
**Marginal Column Totals**	26	11	4	44 (Grand Total)

Forty-four additional unaffected and affected Basset Hounds were selected for confirmatory sequencing of the *RIF1* variant (g.55723957 C->T). The observed total of individuals and percentage of individuals displaying a specific genotype is shown for each cell respectively. The two-tailed P value is 0.57 (Fisher Exact Probability Test)

### Bioinformatic and *In-silico* Analyses

**Fig 6:**
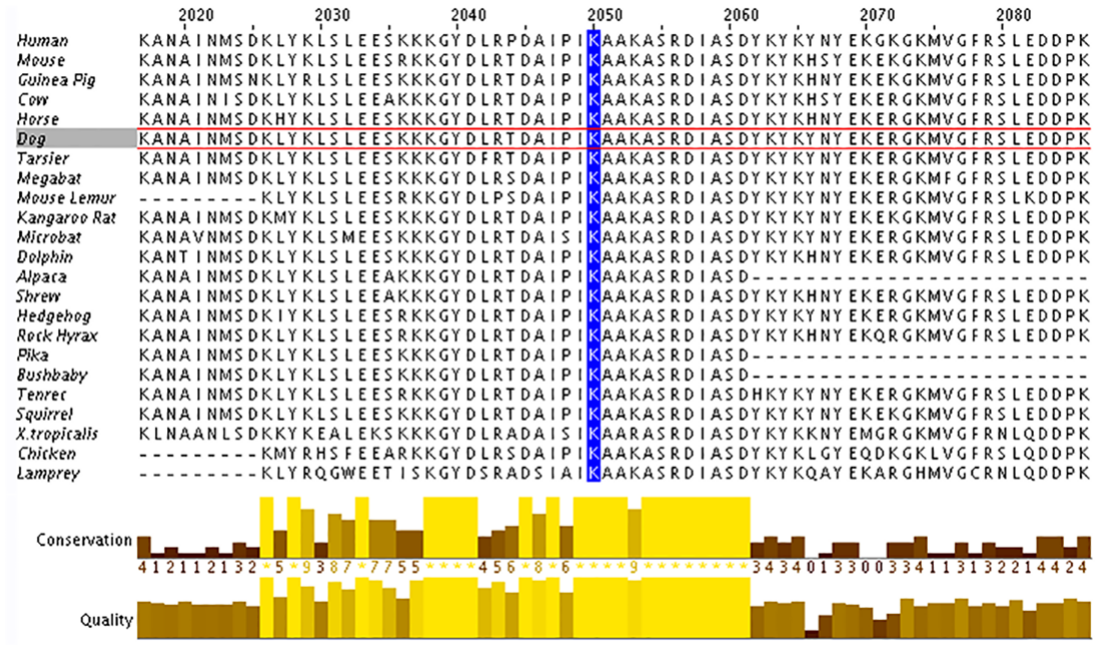
Alignment of the amino acid sequence of exon 48 in Nebulin in several vertebrate species. The Lysine (K) residue (highlighted in blue) at position p.2051 is conserved among 23 vertebrate species.

The Dog Nebulin (*NEB*) resides on chromosome (19q: 55,776,626–55,947,234) and spans 170,609 bp of genomic DNA according to the CanFam2.0 Ensemble Genomic Reference Sequence (http://useast.ensembl.org/index.html). The largest transcript contains 17,721 bp in 128 exons that encode a large 5907 amino acid protein (Transcript ID: ENSCAFT00000009249; Protein ID: ENSCAFP00000008582). Two additional alternatively spliced transcripts have been described for *NEB* in the dog, including (Transcript ID: ENSCAFT00000009252 and ENSCAFT00000009257). Comparison of the 1.82 Mbp PACG-linked canine locus to the human genome revealed synteny to a region on chromosome 2q (Fig 3). The number and order of genes contained within the canine locus are completely conserved in the syntenic human region. Alignment of the amino acid residues demonstrates high genomic conservation, particularly for lysine 2051, among a large number of various vertebrates (Fig 6). *In silico* analysis of the secondary protein structure of Nebulin revealed a large protein consisting of almost 165 Nebulin motifs organized in tandem repeats throughout much of the protein sequence. Each repeat is approximately 35 amino acids long and is predicted to have a α-helical secondary structure that contains a central conserved SXXXY motif. All missense variants identified in Nebulin (K2051R, V2805L and Q2891K) were found to occur in Nebulin motifs.

### Immunohistochemical Analysis of Dog Eyes

**Fig 7:**
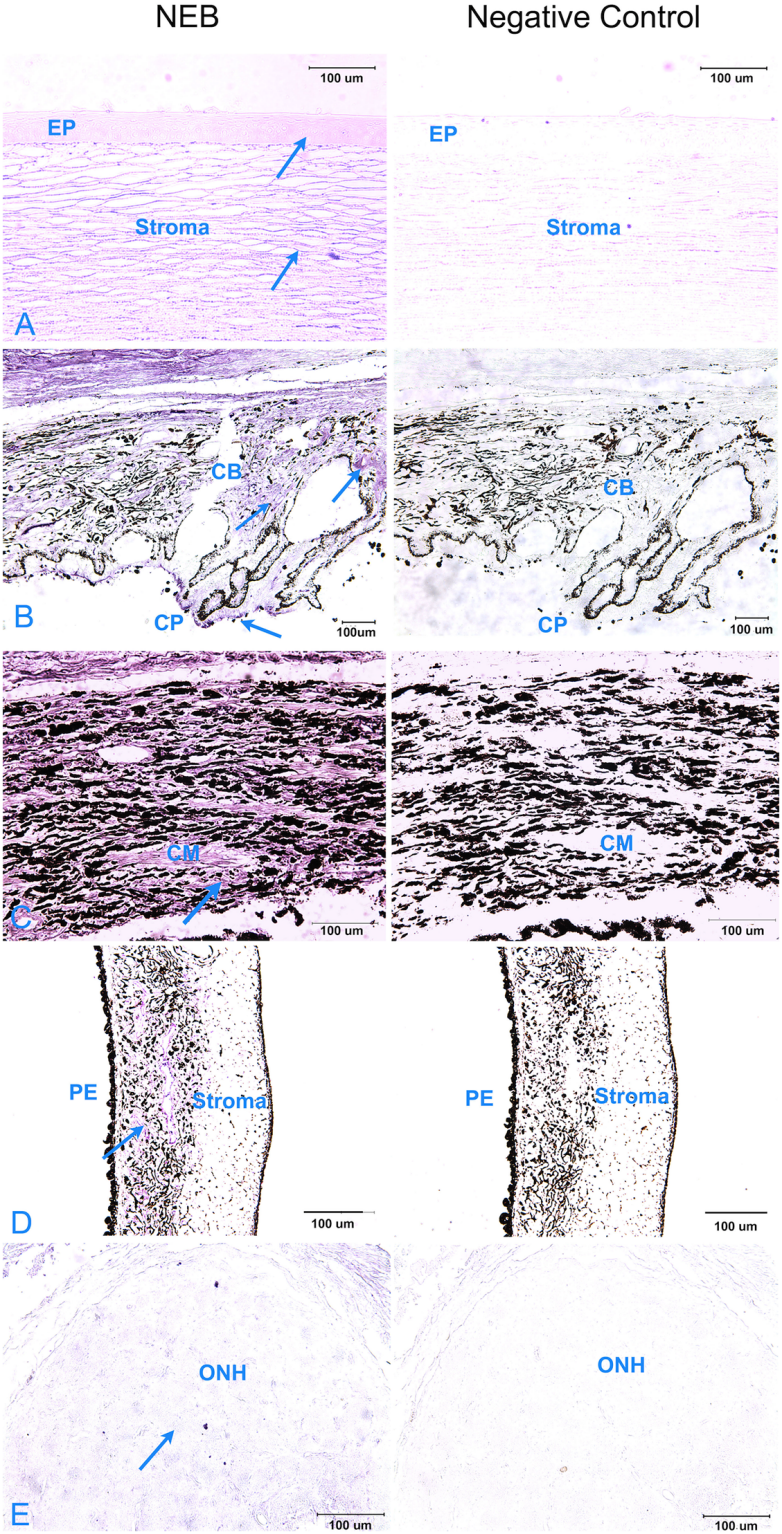
Immunohistochemical localization of Nebulin in sagittal sections of paraffin embedded Basset Hound eyes. Nebulin (left) and negative control (primary antibody omitted) (right) staining are shown. Positive staining indicating protein expression is noted in the stromal and apical epithelial (EP) cell surface of the cornea (Panel A) as well as the ciliary body (CB) and the unpigmented epithelial layer of ciliary processes (CP) (Panel B). Significant expression of Nebulin is observed in the ciliary muscle (CM) (Panel C). Faint, diffused staining is also noted in close proximity to the pigmented epithelium (PE) layer at the apical surface of the iris (Panel D). Faint to no staining was noted in the optic nerve head (Panel E). Arrows indicate areas where staining was observed.

Immunohistochemical assessment of Nebulin in sagittal sections of paraffin embedded dog eyes revealed staining indicative of Nebulin expression in the apical epithelial surface and stromal regions of the cornea (Fig 7A). Diffuse staining was observed throughout the ciliary body and ciliary cleft but was prominent in the unpigmented ciliary epithelial layer that lines the ciliary processes (Fig 7B). Among all ocular substructures, the highest level of Nebulin localization, denoted by intense staining, was observed in the ciliary muscle, further away from the anterior chamber angle (Fig 7C). Faint, diffuse staining was noted in close proximity to the pigmented epithelium at the apical surface of the iris (Fig 7D). Using the methods employed here no staining was noted in the optic nerve head (Fig 7E) or retina (not shown). No obvious differences in Nebulin staining intensity or localization were noted between eyes obtained from affected and unaffected Basset Hounds.

### Immunofluorescence Analysis of Human Donor Eyes

**Fig 8:**
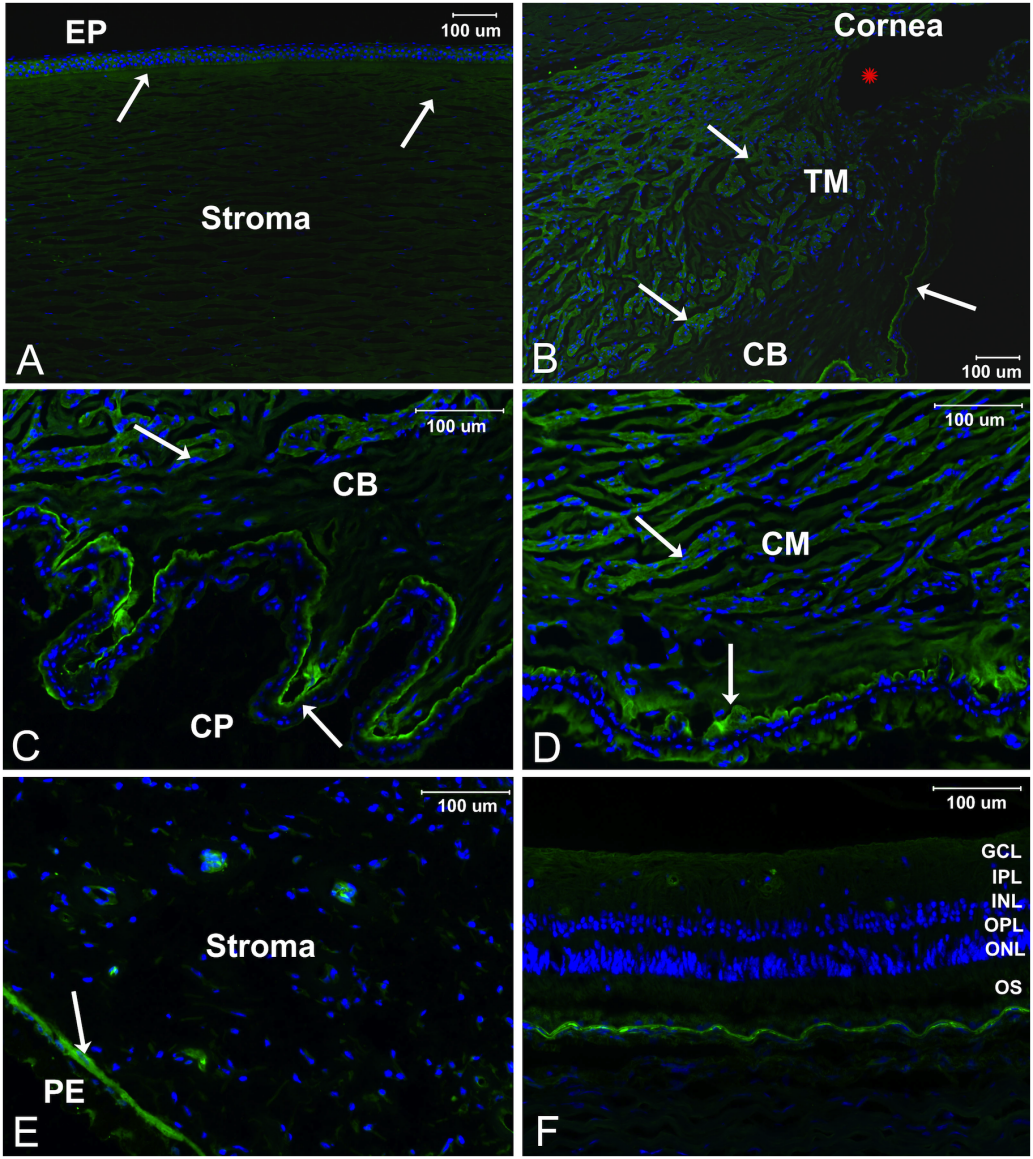
Immunofluorescence localization of Nebulin in in sagittal sections of sucrose embedded human donor eyes. Positive staining indicating protein expression was noted in the stromal and apical epithelial (EP) cell surface of the cornea (A), the trabecular meshwork (TM) and ciliary cleft structure behind the opening of the iridocorneal angle (marked by a red asterisk) (B). Diffuse staining was observed in the ciliary body (CB) and a stronger signal was detected in the unpigmented epithelial layer of ciliary processes (CP) (C). Significant expression of Nebulin denoted by intense staining was observed in the ciliary muscle (CM) (D). Staining was noted in the apical pigmented epithelium (PE) layer of the iris but not within the iris (E). No staining was noted in the retina [OS = outer segment, ONL = outer nuclear layer, OPL = outer plexiform layer, INL = inner nuclear layer, IPL = inner plexiform layer, GCL = ganglion cell layer] (E). Arrows indicate areas where staining was observed.

To confirm the results of Nebulin immunohistochemistry assessment in the dog eye, Nebulin expression was also assessed in human donor eyes using immunofluorescence. Overall, a comparable pattern of Nebulin expression and localization was noted in human eyes when compared to the dog eye. Staining of Nebulin in sagittal sections of sucrose embedded eyes revealed localization in the apical epithelial surface and stromal regions of the cornea (Fig 8A). Prominent staining was noted in the trabecular meshwork and ciliary cleft structures directly behind the iridocorneal angle (Fig 8B). Diffuse staining was noted in the ciliary body, but more prominently in the unpigmented ciliary epithelial layer that lines the ciliary processes (Fig 8C). In concordance with immunohistochemical staining, the highest level of Nebulin localization was observed in the ciliary muscle as well as the apical epithelial layer lining this structure (Fig 8D). With the exception of the apical pigmentary epithelial cell surface and epithelial lining of stromal vessels, no staining was observed within the stromal region of the iris (Fig 8E). No obvious staining was noted in the retina (Fig 8F).

## Discussion

PACG is a complex ocular disease characterized by occlusion of the iridocorneal angle primarily due to miss-positioning of the iris [29] and frequently acute elevation of IOP, resulting in optic nerve damage and acute vision loss. In this study we utilized the Basset Hound as a model to investigate the genetic mechanisms underlying the disease following the identification of a large pedigree with segregating PACG. Investigation of PACG in the Basset Hound revealed a phenotype of slow disease progression, marked by gradual iridocorneal angle collapse in association with elevated IOP, which resembles clinical findings observed in human patients [18,30–32]. The phenotypic similarities of disease presentation in dogs and humans may therefore enable the translation of findings made in this study to human patients with PACG.

Using linkage analysis we identified a 1.82 Mbp locus on chromosome 19q that segregates with PACG in all affected members of a multi-generational Basset Hound pedigree. This finding was refined using homozygosity mapping, which reveals a 0.49 Mbp locus that resides within the PACG-linked region. Exome sequence analysis of the 0.49 Mbp locus revealed four non-synonymous genetic variants that segregate with disease and were further investigated due to their potential for causing functional effects. Of these variants one was located in the gene *RIF1* and three in the gene *NEB*. Only one, the *NEB* variant g.5588214 A->G (p.2051 Lys->Arg), is predicted to result in a possibly pathogenic amino acid substitution within a highly phylogenetically conserved region. However, in this pedigree all non-synonymous variants are in complete linkage disequilibrium.

In order to confirm these findings we determined the allele frequency of *NEB* g.5588214 A->G and *RIF1* variant (g.55723957 C->T) in a second independent Basset Hound cohort more representative of the general population. Data obtained reveal a lack of statistical significance for genotype distribution for *RIF1* (g.55723957 C->T), suggesting that while this variant is in linkage disequilibrium with PACG in our original pedigree, it is not associated with this phenotype in the general Basset Hound population. In contrast, sequence analysis of *NEB* variant g.5588214 A->G demonstrates that homozygocity for the disease-linked allele is clearly associated with PACG (Relative Risk = 3.96, 95% CI 1.4–11.1). However, the disease-associated allele appears to be quite common in the US Basset Hound population and homozygocity for the risk allele was also observed in 33% of the unaffected animals sequenced. Conversely, heterozygocity for the risk allele was observed in a small fraction of the affected dogs.

These data suggest that additional genetic factors are required for the development of the disease. We hypothesize that these additional factors became fixed in the pedigree used for linkage analysis through current breeding techniques, which reduced genetic diversity and resulted in an apparent autosomal recessive pattern of inheritance. The influence of additional genetic factor(s) resulting in complex inheritance and/or incomplete penetrance may also explain the discrepancy between the theoretical maximum LOD_multipoint_ score for the pedigree and the experimentally obtained score of 3.24. While the maximum LOD score statistic for genome-wide linkage is highly robust for gene mapping it is dependent upon the use of accurate assumptions and correct estimation of genetic parameters [33]. Nonetheless, our results suggest that the identified variation has a sufficiently large effect size to cause PACG despite the existence of modifying factors.

Our findings are in accord with previous studies that have indicated that PACG is genetically complex and that multiple loci may contribute to the development of the disease in humans and dogs [34–37]. Our recent findings of two genetically-associated susceptibility loci on chromosome 14 (*COL1A2*) and chromosome 24 (*RAB22A*) in Basset Hounds with PACG also support the notion of genetic complexity in this disease [19]. In this previous study, 3 of the affected animals from the pedigree utilized here were included, but loci on chromosome 19q did not reach genome wide statistical significance. It is conceivable that one or both of these loci interact with the one identified herein, but a larger sample size will be required to obtain statistically meaningful data. Such approaches have been successfully applied to identify genetic loci linked to other forms of the disease [38].

**Fig 9:**
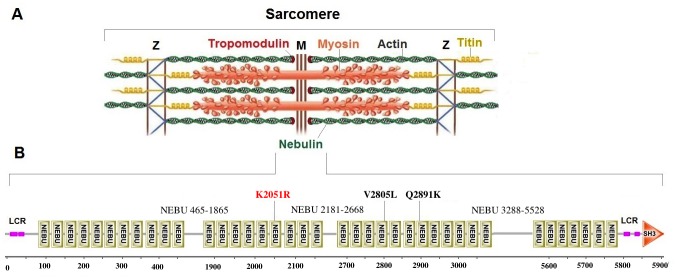
The localization and protein organization of Nebulin within the sarcomere unit of skeletal muscles. Nebulin is a large myofibrillar protein that binds filamentous actin (F-actin) and may also associate with tropomyosin and tropomodulin while anchoring to the z-line of sarcomeres (A). The largest *NEB* transcript is predicted to encode a 5907 amino acid long protein consisting of approximately 165 Nebulin motifs (NEBU). The C-terminus contains a 60 amino acid long SH3 domain. A low complexity region (LCR) was identified at both the N and C-terminals of the protein. The position of the three missense variants identified is indicated (K2051R, V2805L and Q2891K) (B).

The gene *NEB* occupies most of the identified locus and, after exclusion of *RIF1*, contains the only sequence variant predicted to be pathogenic. Nebulin is a large (600–900 kDa), modular protein localized to the thin filaments of sarcomeres in skeletal muscles. It has been characterized as an actin thin filament-binding protein [39]. It is believed that nebulin plays a crucial role in the strict organization of the parallel thin filaments which alignment is crucial for the contractile function of sarcomeres [40]. Due to displaying a length proportional to that of thin filament nebulin may also acts as a thin filament "ruler" that regulates the length and extension of thin filament during sarcomere assembly [39,40]. Evidence supporting its proposed role as a “thin filament ruler” comes from the analysis of its highly modular structure in humans (Fig 9). Additionally the modular organization of Nebulin is crucial for it’s binding to actin and tropomodulin [41–44].

Our immunohistochemistry data indicate that nebulin is expressed in a number of ocular tissues but particularly in the ciliary muscle. This tissue fulfills an important role in the regulation of aqueous humor outflow and regulation of intraocular pressure [45]. Overt differences in the intensity or pattern of immune reactivity were not apparent when eyes of affected and unaffected dogs were examined. We speculate that alterations in nebulin affect the protein’s ability to interact with actin monomers and consequently impact proper assembly and/or contractility of thin filaments. Consequently the robust expression of *NEB* in the ciliary muscle suggests a muscle-related mechanism for the development of PACG in the dog. We hypothesize that the activity of the striated ciliary muscle is required to retain proper positioning of the tissues of the eye’s anterior segment and prevent collapse of the ciliary cleft. This effect may progressively worsen with age as muscle tone decreases in affected animals. Alternatively, it is conceivable that abnormal function of iris muscle is related to abnormal iris volume changes observed in PACG patients [46,47].

This canine locus shares synteny with a region on chromosome 2 in the human genome within which the order and number of genes is conserved. To our knowledge, there have been no reports of *NEB* mutations in association with glaucoma in humans. However, mutations in *NEB* cause some cases of the autosomal recessive disorder nemaline myopathy [48–52]. Patients with nemaline myopathy display a host of phenotypes including muscle weakness and hypotonia [53,54]. Nemaline myopathy due to *NEB* mutations has been found to occur in association with ophthalmoplegia, a condition characterized by weakness or paralysis of one or more extraocular muscles, which are responsible for eye movement [55]. However, it has been suggested that nebulin mutations do not always lead to a severe form of the disorder due to the compensatory role of alternatively spliced isoforms [49]. In Basset Hounds with PACG, an overt systemic phenotype of muscle weakness or hypotonia is not observed. It is possible that the variants we identified in *NEB* resulted in an ocular phenotype by affecting an eye-specific *NEB* isoform. However, we believe it is more likely that these variants result in relatively minor deficits in muscle function that can be tolerated outside of the narrow spatial confines of the ocular anterior segment.

Investigation of the homeostatic role of nebulin in maintaining the ciliary cleft structure and facilitating aqueous humor outflow can provide new insight into the physiological mechanisms of normal eye function as well as mechanisms underlying the development of PACG. It is our hope that our findings will help unravel the molecular mechanisms and genetic pathways that contribute to PACG development in dogs as well as patients with the disease.
